# Li_1.2_Mn_0.54_Ni_0.13_Co_0.13_O_2_-Encapsulated Carbon Nanofiber Network Cathodes with Improved Stability and Rate Capability for Li-ion Batteries

**DOI:** 10.1038/srep11257

**Published:** 2015-06-08

**Authors:** Dingtao Ma, Peixin Zhang, Yongliang Li, Xiangzhong Ren

**Affiliations:** 1 School of Chemistry and Chemical Engineering, Shenzhen University, Shenzhen, Guangdong, 518060, PR China

## Abstract

Li_1.2_Mn_0.54_Ni_0.13_Co_0.13_O_2_-encapsulated carbon nanofiber network cathode materials were synthesized by a facile electrospinning method. The microstructures, morphologies and electrochemical properties are characterized by X-ray diffraction (XRD), field-emission scanning electron microscopy (FE-SEM), high resolution transmission electron microscopy (HR-TEM), galvonostatic charge/discharge tests, cyclic voltammetry and electrochemical impedance spectroscopy (EIS), etc. The nanofiber decorated Li_1.2_Mn_0.54_Ni_0.13_Co_0.13_O_2_ electrode demonstrated higher coulombic efficiency of 83.5%, and discharge capacity of 263.7 mAh g^−1^ at 1 C as well as higher stability compared to the pristine particle counterpart. The superior electrochemical performance results from the novel network structure which provides fast transport channels for electrons and lithium ions and the outer carbon acts a protection layer which prevents the inner oxides from reacting with HF in the electrolyte during charge-discharge cycling.

LiCoO_2_ has been widely applied as one of the commercial cathode materials for Li-ion batteries since its discovery since 1992. However, the relatively low capacity (140 mAh g^−1^), high cost and toxicity of the cobalt have hindered its further application for the demand of future electric technology. In this case, development of alternative higher energy, lower cost and environment friendly cathodes is critical for next generation Li-ion batteries^1^,^2^.

Layer structure LiMO_2_ (M = Mn, Co, Ni, etc), spinel LiMn_2_O_4_ and olivine LiFePO_4_ are also regarded as the main candidate cathodes in recent years. Among them, Li-rich cathode materials xLi_2_MnO_3_ • (1-x)LiMO_2_ (M = Mn, Co, Ni) have drawn much attention owing to its high capacity of more than 250 mAh g^−1^ at a lower cost compared to LiCoO_2_. Despite these merits, Li-rich layered oxides always suffer from the inferior cycling stability and rate capability, which impede their applications^1^,^3^. Surface modification^4^,^5^,^6^,^7^,^8^, doping strategy^9^,^10^,^11^,^12^, and forming 3D porous structure^13^ are three common approaches introduced to improve their electrochemical performances. As an effective and inexpensive way of truly producing one dimensional (1D) fibers from micrometer to nanometer size ranges in diameter, electrospinning^14^,^15^ has been widely used in many areas, including the fabrication of electrode materials, especially the anode^16^,^17^,^18^,^19^ and cathode^20^,^21^,^22^,^23^,^24^ materials for Li-ion batteries.

Herein, for the first time, Li_1.2_Mn_0.54_Ni_0.13_Co_0.13_O_2_-encapuslated carbon nanofiber network cathodes were prepared by the combination of electrospinning and heat treatment. For comparison, pristine Li_1.2_Mn_0.54_Ni_0.13_Co_0.13_O_2_ particles were also synthesized by sol-gel method. It was demonstrated that the nanofiber decorated Li_1.2_Mn_0.54_Ni_0.13_Co_0.13_O_2_ composites displayed higher capacity retention, rate capability and more stable cyclic performance compared to the pristine counterpart. The results not only give a further insight into the reaction mechanism but also provide a rational direction to synthesize cathode materials for Li-ion batteries.

## Results and Discussion

**Figure 1:**
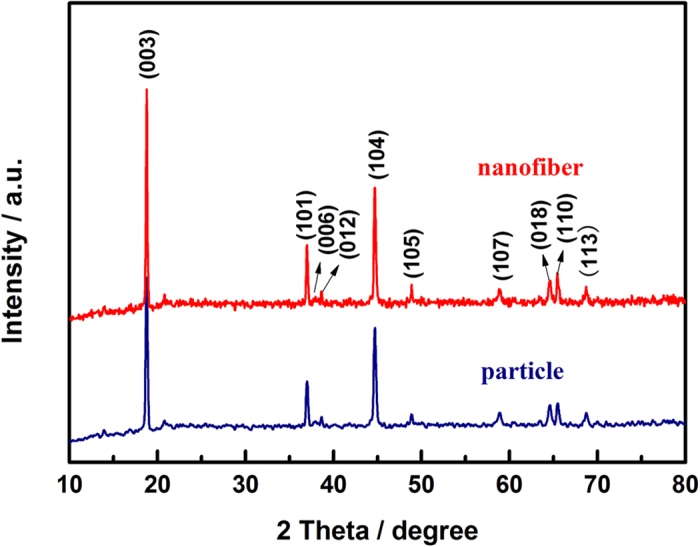
XRD patterns of the particle and nanofiber decorated Li_1.2_Mn_0.54_Ni_0.13_Co_0.13_O_2_ samples.

Figure 1 shows the XRD patterns of the pristine particle and nanofiber decorated samples. The pristine particle sample shows the α-NaFeO_2_ structure without any impurity reflections. The weak superstructure reflections around 2θ = 20°–25° belongs to the ordering of the transition metal and Li ions in the transition metal layer of the lattice^25^,^26^. Clear separation of adjacent peaks of (006)/(012) and (108)/(110) indicates that the samples have a well crystalline layered structure^27^,^28^,^29^. For the nanofiber decorated sample, no significant lattice parameter differences were recognized in the main reflections corresponding to the α-NaFeO_2_ structure, and no additional reflections corresponding to carbon are observed due to its amorphous structure^21^,^22^.

**Figure 2:**
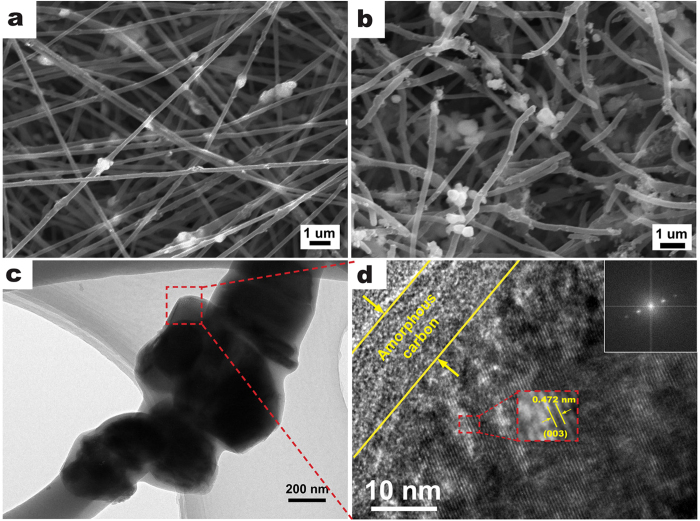
SEM (**a**,**b**) and HRTEM (**c**,**d**) images of nanofiber decorated Li_1.2_Mn_0.54_Ni_0.13_Co_0.13_O_2_ samples before (**a**) and after (**b**–**d**) heat treatment. The inset of d is the SAED pattern.

The morphologies and strucutres of the nanofiber decorated sample were shown in Fig. 2. Compared to the pristine particles (Supporting Information, Figure 1S), which showed a universal and slight agglomeration with a uniform particle size ranging between 300 and 500 nm, the carbon nanofiber contained Li_1.2_Mn_0.54_Ni_0.13_Co_0.13_O_2_ particles which were evenly dispersed with carbon black, excepting a little agglomeration after electrospinning (Fig. 2a). The morphology of nanofiber decorated Li_1.2_Mn_0.54_Ni_0.13_Co_0.13_O_2_ after heat treatment in air was shown in Fig. 2b. The nanofibers shrinked and partly broken, some particles were inevitably squeezed out from the nanofibers and then distributed along the surface or junction of the nanofibers after agglomeration. Figure 2c,d show the HRTEM images of the samples. Result (Fig. 2c) confirms that Li_1.2_Mn_0.54_Ni_0.13_Co_0.13_O_2_ particles and carbon black are dispersed inside the carbon nanofiber matrix. The particles exhibit continuous interference fringe spacing of around 0.47 nm corresponding to the (003) lattice fringes of the hexagonal layered structure (Fig. 2d)^3^,^6^,^28^,^29^. In addition, amorphous carbon layers with a thickness about 10 nm were observed on the surface of the Li_1.2_Mn_0.54_Ni_0.13_Co_0.13_O_2_ particles, which may be regarded as protection layer during electrochemical cycling. The selected area electron diffraction (SAED) pattern (inset of Fig. 2d) confirms the single crystallinity for particles.

**Figure 3:**
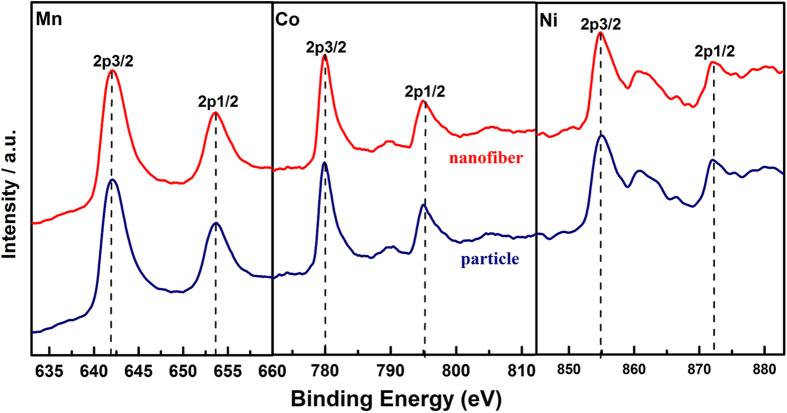
XPS spectra of nanofiber decorated and particle Li_1.2_Mn_0.54_Ni_0.13_Co_0.13_O_2_ samples before and after heat treatment.

X-ray photoelectron spectroscopy (XPS) measurements were used to investigate the variation in chemical states of Mn, Ni and Co elements. Figure 3 shows typical XPS spectra for Mn2p, Co2p and Ni2p for the pristine and nanofiber decorated samples. For these two samples, the observed binding energies of Mn2p, Co2p and Ni2p remain at 642, 780 and 855 eV, which coincide well with those for Mn^4+^, Co^3+^ and Ni^2+^, respectively^30^,^31^. Therefore, the valences of Mn, Co and Ni did not change after the heat treatment with/without electrospinning.

**Figure 4:**
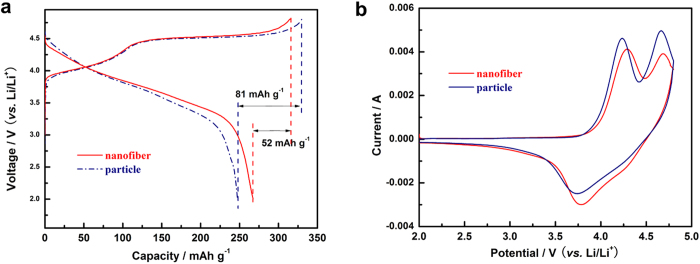
Charge-discharge profiles (**a**) at 0.2 C and CV curves (**b**) scanned at 1 mV s^−1^ of particle and nanofiber decorated Li_1.2_Mn_0.54_Ni_0.13_Co_0.13_O_2_ electrodes.

The first charge-discharge curves during electrochemical cycling of the two cathode materials taken at 0.2 C, are shown in Fig. 4a. During the charging process, both samples exhibit two distinguishable stages, a smoothly sloping voltage profile below 4.5 V corresponding to Li^+^ deintercalation from LiMn_1/3_Ni_1/3_Co_1/3_O_2_ component and a long plateau around 4.5 V related to Li^+^ and O^2−^ extracted from the Li_2_MnO_3_ phase and structural rearrangement^1^,^31^,^32^,^33^. The first discharge capacity of nanofiber decorated Li_1.2_Mn_0.54_Ni_0.13_Co_0.13_O_2_/C and pristine sample are 263.7 and 247.2 mAh g^−1^, with the coulombic efficiency of 83.5% and 75.4%, respectively. The higher coulombic efficiency and discharge capacity may be attributed to the reasons as follow: (a) the amorphous carbon layer prevents the formation of inactive O_2_ molecules and the side reactions of electrolyte; (b) the better dispersity and less agglomeration of the cathode particles would increase the active surface area and facilitate the electrons and/or ions migration during cycling.

Figure 4b shows the CV profiles of the first cycle of the two samples at a sweep rate of 1 mV s^−1^ between 2.0–4.8 V. As can be seen, the initial anodic peaks of the samples at about 4.2 V are due to the lithium extraction from the Li_1.2_Mn_0.54_Ni_0.13_Co_0.13_O_2_ lattice and the oxidation process of Ni^2+^/Co^3+^ to higher oxidation states^2^,^30^,^34^,^35^,^36^, while the sole cathodic peak occurs at around 3.8 V is attributed to the reduction of Ni^2+^/Co^3+^. The second anodic peaks located in the potential of 4.6 V are normally associated with the oxygen release process from Li_2_MnO_3_-type component as well as the electrolyte oxidation. Compared with the CV curve of pristine Li_1.2_Mn_0.54_Ni_0.13_Co_0.13_O_2_, the decreased intensity and area of anodic peak around 4.6 V of the nanofiber decorated Li_1.2_Mn_0.54_Ni_0.13_Co_0.13_O_2_ sample implies that the O_2_ release was greatly suppressed by the amorphous carbon layers and more available Li^+^ sites were remained. This agrees with the improved columbic efficiency of the composite sample (Fig. 4a).

**Figure 5:**
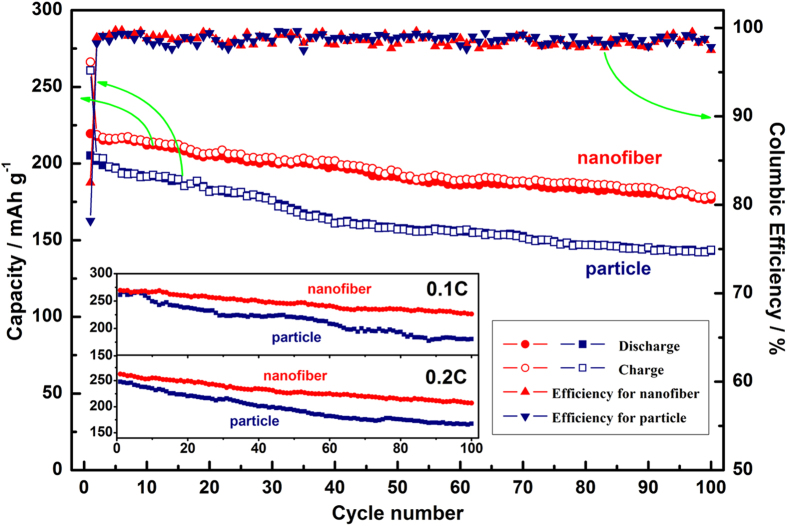
Cyclic performance and coulombic efficiency of particle and nanofiber decorated Li_1.2_Mn_0.54_Ni_0.13_Co_0.13_O_2_ samples at 1 C. The inset is the discharge capacities of two samples at 0.1 and 0.2 C.

The cyclic performances of both samples at 1 C are shown in Fig. 5. The first discharge capacity of nanofiber decorated Li_1.2_Mn_0.54_Ni_0.13_Co_0.13_O_2_ and particle samples are 219.5 and 205 mAh g^−1^ with a coulombic efficiency of 82.5% and 76.2%, respectively. After 100 cycles, the nanofiber samples still display a discharge capacity of 176.7 mAh g^−1^ with a capacity retention of 80.5%, while the particle samples only show a discharge capacity of 145.9 mAh g^−1^ and a capacity retention of 71.1%. Their cyclic stability at low rate of 0.1 and 0.2 C are also shown in the inset of Fig. 5. The two samples deliver a similar discharge capacity in the first several cycles. However, the nanofiber samples still retain a higher discharge capacity than the pristine one both at 0.1 and 0.2 C after 100 cyles. The improved cyclic stability and capacity retention of the nanofiber cathodes may be attributed to the synergistic effect of the larger specific surface area and the protection of coating layer. Comparing with the pristine samples, the larger specific surface area of nanofibers provides more transportation paths for ions and/or electrons, and the coating layer simultaneously prevents the inner oxide from reacting with HF in the electrolyte and twice agglomeration during electrochemical test.

**Figure 6:**
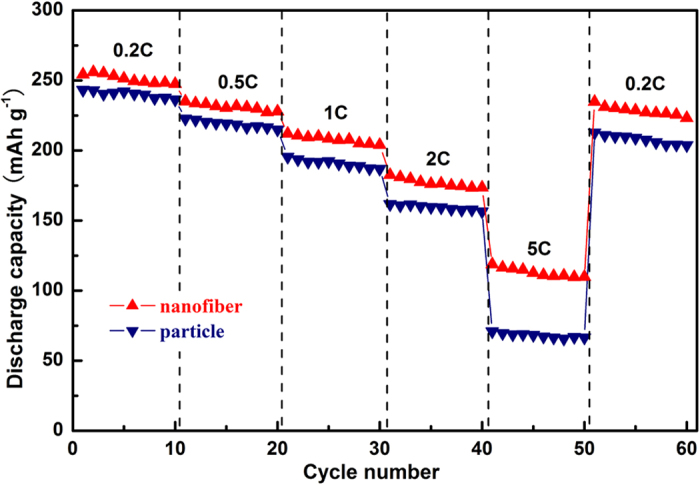
Cycling behavior of particle and nanofiber-decorated Li_1.2_Mn_0.54_Ni_0.13_Co_0.13_O_2_ samples at various rates.

Figure 6 shows a continuous cycling result at incremental rates from 0.2 to 5 C then recovering back to 0.2 C. Similar discharge capacity is observed at lower rate for both samples, but obvious capacity differences increase gradually with the increasing rate. As can be seen, the rate capability for nanofiber decorated Li_1.2_Mn_0.54_Ni_0.13_Co_0.13_O_2_ samples has been significantly enhanced compared to the particle. Such contrast observation might be related to the faster ionic and/or electronic diffusion and shorter migration path at high rate, which were benefited from the network structures.

**Figure 7:**
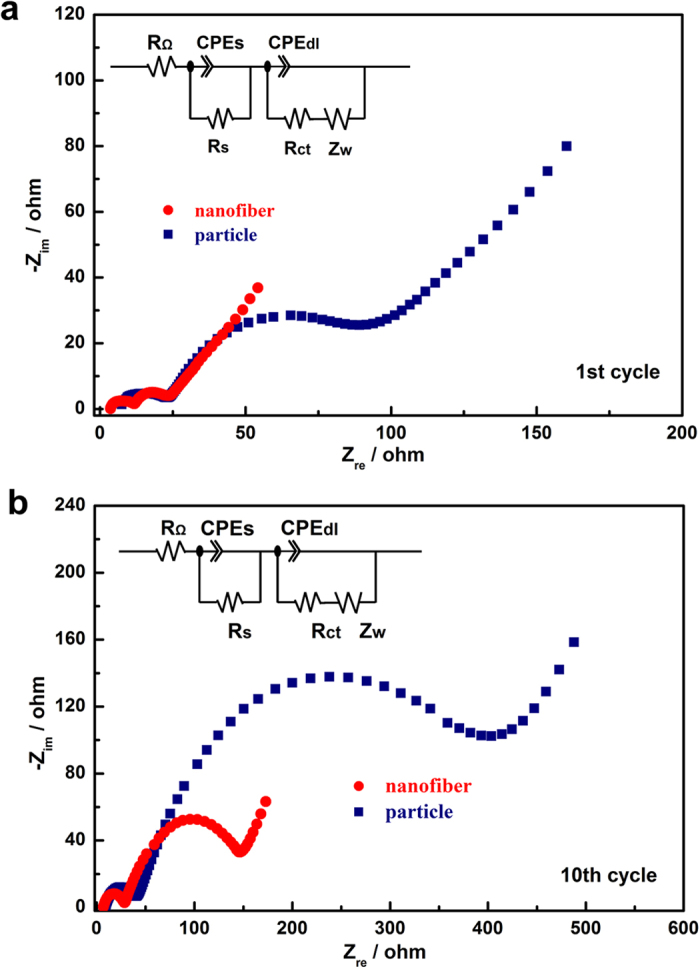
EIS plots of particle and nanofiber decorated Li_1.2_Mn_0.54_Ni_0.13_Co_0.13_O_2_ samples after 1st (**a**) and 10th (**b**) cycle at a same state of discharge at 3.5 V. The insets of a and b are the equivalent circuits.

To investigate the origin of the improved electrochemical performance of nanofiber decorated Li_1.2_Mn_0.54_Ni_0.13_Co_0.13_O_2_ sample, EIS plots of both samples were collected. Figure 7 compares the EIS spectra of the particle and nanofiber samples at different states of cycling, 1st and 10th. An equivalent circuit (inset of Fig. 7)^28^ was applied to fit the raw data from EIS measurements to obtain the accurate values of different resistances. In this equivalent circuit, R_Ω_ is the resistance of the solution , R_s_ is the resistance of ion diffusion in the region of the surface layer of particle, R_ct_ represents the charge transfer resistance in the electrode/electrolyte interface and Z_w_ refers to Warburg impedance .The fitting values of R_s_ and R_ct_ were summarized in Table 1.

Compared with the Fig. 7a,b, there was a huge reduction of R_ct_ for the nanofiber sample upon cycling. After 10 cycles, the R_ct_ values of nanofiber and particle samples increased from 21.73 and 30.91 Ω to 118.26 and 363.31 Ω, respectively. The reduction of R_ct_ for nanofiber sample may facilitate lithium transfer on the electrode/electrolyte interface, then leads to improved rate capability. Therefore, the better electrochemical performance of nanofiber sample compared to the particle one can be ascribed to larger surface area and better electrical contact of the particle, which increase the active specific surface area and decrease the polarization effect.

## Conclusions

In conclusion, Li_1.2_Mn_0.54_Ni_0.13_Co_0.13_O_2_-ecapulated carbon nanofiber network were prepared through a facile electrospinning method following by a heat treatment. XPS spectra demonstrate that the valence of Mn, Ni, Co did not change after the heat treatment. Compared with the pristine Li_1.2_Mn_0.54_Ni_0.13_Co_0.13_O_2_ particles, the nanofiber sample has the obviously improved electrochemical performance. The coulombic efficiency of the first charge-discharge process is enhanced from 76.2% to 82.5% with a capacity retention of 80.5% at 1 C after 100 cycles. In addition, the nanofiber sample also exhibits superior rate capability compared to the pristine one. Such improved initial coulombic efficiency, cyclic stability and rate capability can be attributed to the suppression of the side reaction of electrolyte, larger surface area of nanofibers, better dispersity of active particles and more importantly, faster electron and ion transportation, which are benefited from the carbon nanofiber network. Therefore, it is believed that the nanofiber decorated Li_1.2_Mn_0.54_Ni_0.13_Co_0.13_O_2_ should be one of ideal cathode candidates and the results also provide a rational direction to synthesize other cathode materials for Li-ion batteries.

## Methods

### **Preparation of Li**_**1.2**_ **Mn**_**0.54**_ **Ni**_**0.13**_ **Co**_**0.13**_ **O**_**2**_ **particles**

Li_1.2_Mn_0.54_Ni_0.13_Co_0.13_O_2_ particles were prepared by a PVP-assisted sol-gel method. Typically, stoichiometric amounts of Li(CH_3_COO)∙2H_2_O, Mn(CH3COO)_2_∙4H_2_O , Ni(CH_3_COO)_2_∙4H_2_O and Co(CH_3_COO)_2_∙4H_2_O were first dissolved in 10 wt% polyvinylpyrrolidone (PVP)/H_2_O mixed solution under vigorous stirring for 6 hours to form a homogeneous solution. Then the solution was evaporated at 85 °C until a viscous violet gel emerged. The gel was dried in a vacuum oven at 120 °C for 18 h. The precursor powders were decomposed at 500 °C for 6 h to guarantee the complete decomposition of (PVP). After a grinding for 15 min with a mortar and pestle, the obtained powders were calcined at 950 °C for 8 h in air and ground again for at least 20 min to get the finial products.

### **Fabrication of Li**_**1.2**_ **Mn**_**0.54**_ **Ni**_**0.13**_ **Co**_**0.13**_ **O**_**2**_ **/C composite fibers**

After milling for 1 h, the mixture of 30 wt% of Li_1.2_Mn_0.54_Ni_0.13_Co_0.13_O_2_ particles and 8 wt% of carbon black were added into the 8 wt% polyacrylonitrile (PAN)/dimethyl formamide (DMF) solution and then stirred with ultrasonic dispersion for at least 24 h. Electrospinning was carried out with 18 KV high voltage, 1 ml/h feeding rate and 20 cm needle-to-collector distance, respectively. The electrospun nanofibers were collected on the aluminum foil and dried in a vacuum oven at 120 °C for 12h. The obtained electrospun nanofibers were firstly heated at 280 °C for 1 h with a heating rate of 5 °C min^−1^, then at 350 °C for 1 h and finally at 400 °C for 1 h with a heating rate of 2 °C min^−1^ to form Li_1.2_Mn_0.54_Ni_0.13_Co_0.13_O_2_-encapuslated carbon nanofibers. In order to have a fair comparison, the pristine Li_1.2_Mn_0.54_Ni_0.13_Co_0.13_O_2_ particles were also having the same heat treatment without electrospinning. The carbon content of nanofiber decorated Li_1.2_Mn_0.54_Ni_0.13_Co_0.13_O_2_ was evaluated by elemental analysis to be around 10.57%–10.82%. Additionally, the specific surface areas of the particles and nanofibers were 4.2 m^2^/g and 28.56 m^2^/g, respectively, which were tested by using NOVA1200e (Quantachrome).

### Structure and morphology characterization

X-ray diffraction (XRD) (Bruker D8 Advance) with Cu Kα radiation operated at 40 KV and 40 mA was employed to identify the crystal structure (2θ = 10°~80°). Scanning electron microscope (SEM) (Hitachi, S-3400N) and high resolution transmission electron microscope (HRTEM) (Tecnai G2 F30) were used to observe the morphologies and structures. X-ray photoelectron spectroscopy (ESCALAB 250Xi) was used to test the chemical state of Mn, Ni and Co of the samples. With regard to the HRTEM samples preparation, a trace of nanofiber decorated Li_1.2_Mn_0.54_Ni_0.13_Co_0.13_O_2_ sample were added into ethanol solution with ultrasonic dispersion for 7 h, then the testing liquid was dropped to the micro Cu grid and heated at 60 °C for 1 h.

### Electrochemical measurements

Electrochemical tests were made using CR-2032 coin cells, which were assembled in a glove box (MBRAUN,UNILab2000) filled with high purity argon, comprising cathode, Li metal anode and a polymer separator (Celgard 2400) with 1 M LiPF_6_ in EC:DMC (1:1 in volume ratio) as electrolyte. For fabrication of the cathodes, nanofiber decorated Li_1.2_Mn_0.54_Ni_0.13_Co_0.13_O_2_ was mixed with polyvinylidene fluoride (PVDF) with a weight ratio of 95:5 in N-methyl-2-pyrrolidone (NMP), while pristine Li_1.2_Mn_0.54_Ni_0.13_Co_0.13_O_2_ particles were mixed with acetylene black and PVDF with a weight ratio of 85:10:5. The obtained slurry was coated onto Al foil and roll-pressed. The electrodes were dried overnight at 120 °C in vacuum oven. Cyclic performance and rate capability tests were conducted on a battery station (LAND, CT2001A) between voltage range of 2.0–4.8 V (1 C = 250 mA g^−1^). Both of the Cyclic voltammetry (CV) and electrochemical impedance spectroscopy (EIS) (at a frequency range of 10^−2^–10^5^ Hz) were conducted using an electrochemical workstation (CHI660A), and the CV was applied with a scan rate of 1 mV/s between 2.0–4.8 V. All of the electrochemical tests were carried out at room temperature.

## Additional Information

**How to cite this article**: Ma, D. *et al.* Li1.2Mn0.54Ni0.13Co0.13O2-Encapsulated Carbon Nanofiber Network Cathodes with Improved Stability and Rate Capability for Li-ion Batteries. *Sci. Rep.* **5**, 11257; doi: 10.1038/srep11257 (2015).

## Supplementary Material

Supplementary Information

## Figures and Tables

**Table 1:** **Fitting values of R**_**s**_ **and R**_**ct**_ **of the particle and decorated samples at the 1st and 10th cycling.**

	**R**_**s**_**/Ω**		**R**_**ct**_**/Ω**	
	**1st**	**10th**	**1st**	**10th**
Nanofiber	8.21	11.65	21.73	118.26
Particle	15.46	62.61	30.91	363.31
