# Supplementary material for: Li1.2Mn0.54Ni0.13Co0.13O2-Encapsulated Carbon Nanofiber Network Cathodes with Improved Stability and Rate Capability for Li-ion Batteries
Source: Sci Rep. 2015 Jun 8;5:11257. doi: 10.1038/srep11257 (PMC4459222; doi:10.1038/srep11257)
Supplement: Supplementary Information [file srep11257-s1.doc]

**Li_1.2_Mn_0.54_Ni_0.13_Co_0.13_O_2_-Encapsulated Carbon Nanofiber Network Cathodes with Improved Stability and Rate Capability for Li-ion Batteries**

Dingtao Ma, Peixin Zhang,* Yongliang Li,* Xiangzhong Ren

School of Chemistry and Chemical Engineering, Shenzhen University, Shenzhen, Guangdong, 518060, PR China

* Corresponding author: Peixin Zhang, Tel & Fax: 86-755-26558134, Email: pxzhang@szu.edu.cn

Yongliang Li, Tel: 86-755-26536627, Email: liyli@szu.edu.cn


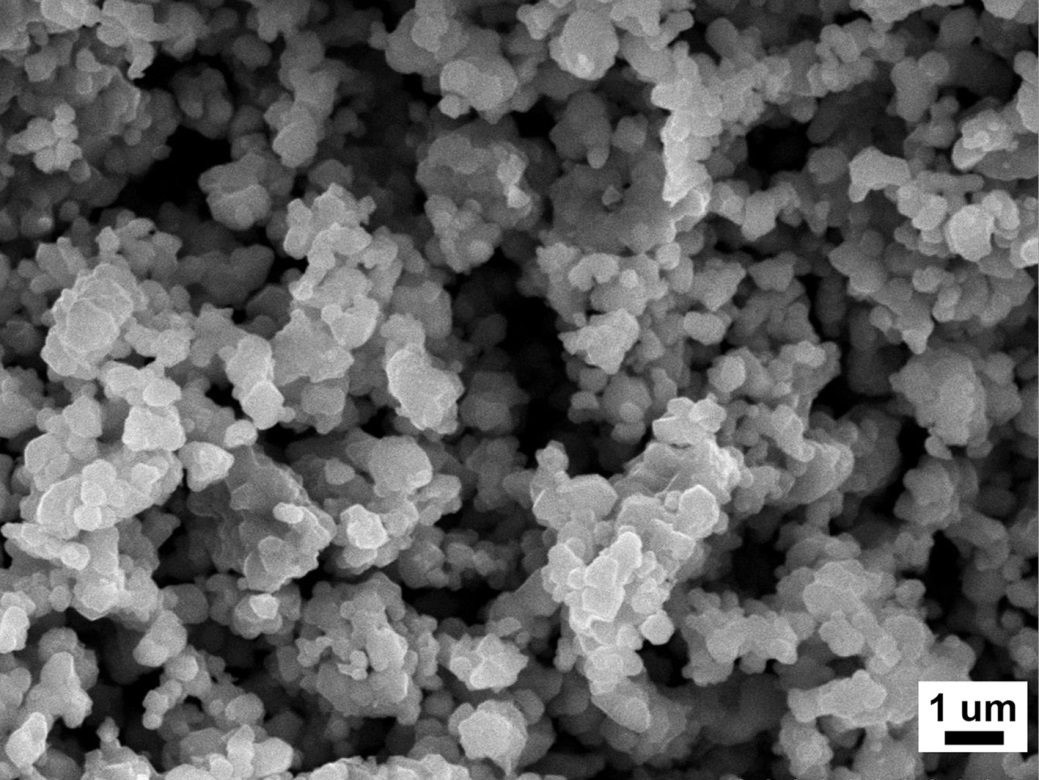


**Figure S1** SEM image of Li_1.2_Mn_0.54_Ni_0.13_Co_0.13_O_2_ particles.
